# Simulations for determining the optimal enhancement ratio threshold for segmentation of left atrial fibrosis

**DOI:** 10.1186/1532-429X-17-S1-P60

**Published:** 2015-02-03

**Authors:** Dana C Peters, Litten K Bertelsen

**Affiliations:** 1 Diagnostic Radiology, Yale School of Medicine, New Haven, CT, USA; 2 Cardiology, Copenhagen University Hospital, Copenhagen, Denmark

## Background

Segmentation of enhanced signal on late gadolinium enhancement (LGE) images of the eft atrium (LA) is required for characterizing left atrial fibrosis. However, the analysis is challenging, because the atrial fibrosis is not readily visually apparent. Recently, a study advocated using enhancement ratio (ER, atrial wall to blood) to segment the atrium, and found that an ER of 1.6 corresponded to very low voltage on EP mapping (<0.1mV) (1). Use of ER for thresholding is attractive, because thresholds based on CNR values (typically 3.5) or visually chosen are sensitive to underlying SNR. We sought to investigate the capabilities of ER for segmentation, using simulations.

## Methods

Simulations using Bloch equations modeled the signal intensity of an 1RR inversion recovery spoiled gradient echo sequence, with inputs of TR, θ, constant RR interval, views per segment (vps), TI, and T1, assuming centric order. All simulations used typical values of TR=4.4ms, 33 vps, θ=15deg, 1000 ms RR, and TI set to null normal myocardium. Experiments were performed on a 1.5T Siemens using T1 standards, and typical protocol parameters to confirm simulations with respect to ER. Next, for a range of expected blood T1 (T1_b_) values (300 to 370ms, at 20 to 50 minutes post injection) (2), the atrial myocardium T1 (T1_a_) values were calculated. T1_a_ was calculated for each extra-cellular volume fraction (ECV) value of interest (2), using T1_b_0 of 1500ms and T1_a_0 of 900ms, with R1=4.5 L/mMol/s and hematocrit HCT=0.46. The T1_a_ and T1_b_ were input into the validated Bloch simulations to calculate the ER.

## Results

**Figure 1:**
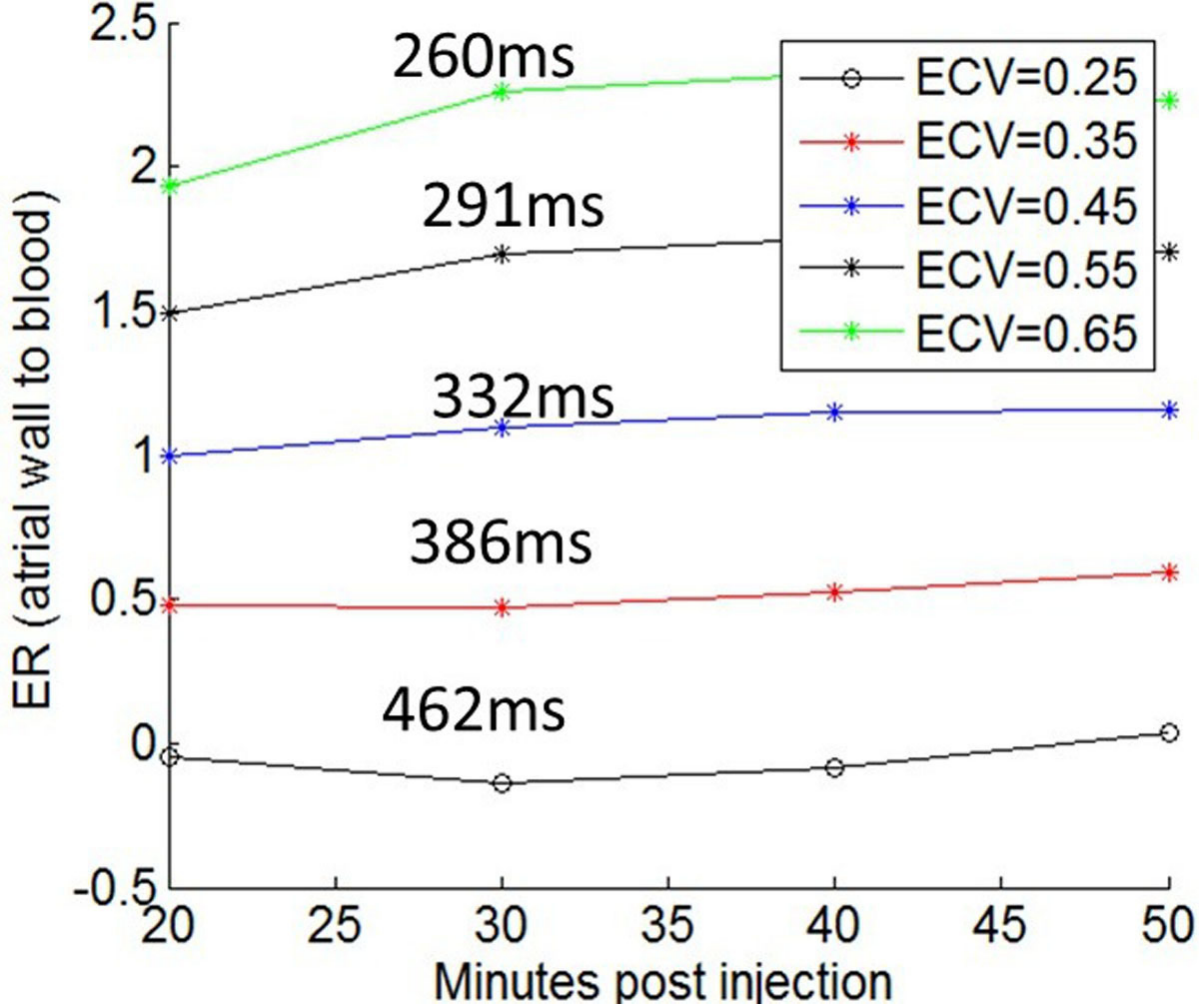
Atrial wall-blood enhancement ratio (ER) is plotted vs. minutes post injection for 0.2 mmol/kg Gd-DTPA, for multiple atrial wall extracellular volume fractions (ECV). T1_b_ was simulated as 300, 340, 360 and 370ms for the 20 to 50 minutes time-points. The T1_a_ values at 30 minutes are shown.

**Figure 2:**
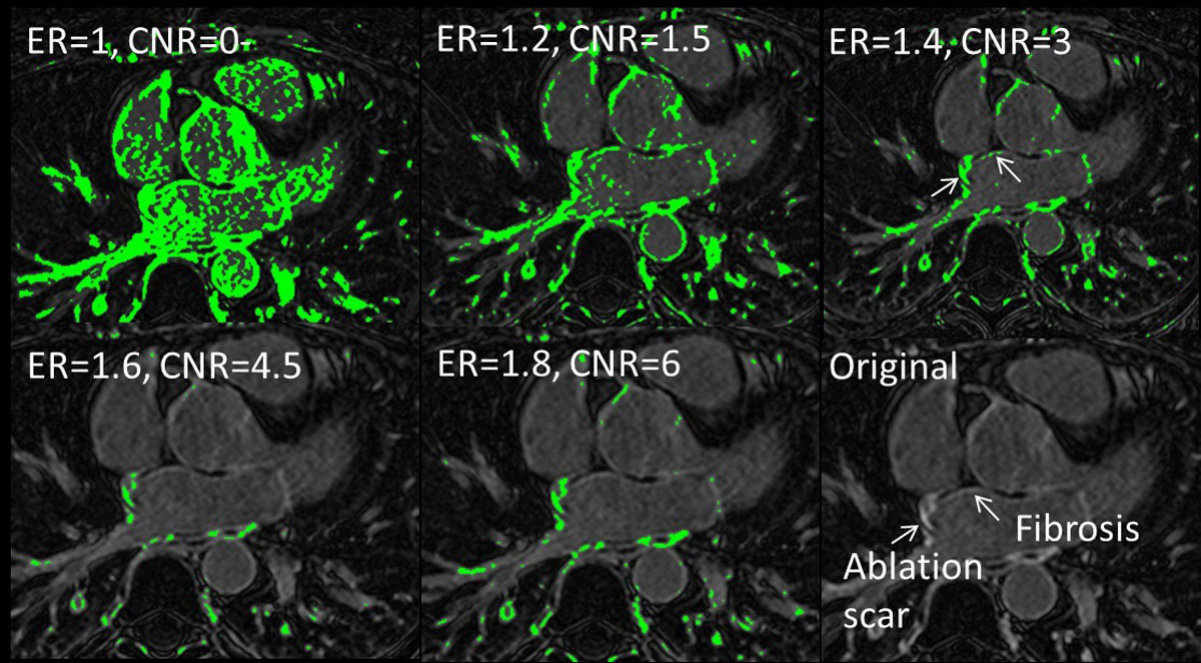
Example of using ER to segment LGE images.

Comparing experimental and simulated ERs for a range of T1s and imaging protocols, we found good agreement. Figure 1 plots ER, for ECV values from 0.25 (normal myocardium) to 0.7 (abject scar). Simulations show ER is not strongly dependent on heart-rate but is dependent on nulling. Note that imaging later post injection is helpful. Also note that partially fibrotic tissue (i.e. ECV >0.5) can be visualized, with an ER >1.3. Figure 2 shows the results of segmenting an LGE slice, which has ablation scar and atrial fibrosis. In these segmentations, CNR and ER are given. Figure 2 demonstrates that an ER threshold of 1.4 includes both atrial fibrosis and scar.

## Conclusions

These simulations find that an ER > 1.6 indicates an ECV > 0.55, which corresponds to very fibrotic myocardium, in agreement with Khurram et al. Furthermore, an ER of 1.4 is a practical threshold which permits segmentation of partially fibrotic tissue.

## Funding

We gratefully acknowledge support from NHLBI R01HL113352.
